# Development of a Piezoelectric Immunosensor to Rapidly Measure Prohibitin‑1 as a Prognostic Biomarker for Sepsis and Other Serious Infectious Diseases

**DOI:** 10.1021/acsaelm.5c01783

**Published:** 2025-12-02

**Authors:** Eman S. Kamel, Yusra Rahman, Jolonda C. Mahoney, Ethan J. Anderson, Reza Nejadnik

**Affiliations:** Department of Pharmaceutical Sciences and Experimental Therapeutics, College of Pharmacy, 4083 University of Iowa, Iowa City, Iowa 52242, United States

**Keywords:** quartz crystal microbalance, immunosensor, sepsis, prohibitin-1, biomarker detection, MODS, frequency shift, ELISA

## Abstract

Sepsis is a life-threatening condition and the leading cause of mortality worldwide, particularly when an affected patient develops multiple organ dysfunction syndrome (MODS), a serious complication defined as progressive and potentially irreversible dysfunction in two or more organ systems. Clinical and experimental studies strongly suggest that patient outcomes with sepsis would improve if the risk for MODS and mortality could be identified in the first few hours after the onset of symptoms. We present an approach leveraging quartz crystal microbalance with dissipation monitoring (QCM-D) technology for rapid (∼30 min) detection of prohibitin-1 (PHB1), a protein with pleiotropic biological function that dramatically increases in blood during the acute phase of an infection. The feasibility of detection and reproducible quantification of recombinant PHB1 in physiological buffered conditions is described. Sensitivity and detection limit for the third overtone (12.19 Hz μg^–1^ mL and 59.06 ng/mL, respectively) were determined to be within the physiological range, highlighting the QCM-D immunosensor’s potential for applied use in plasma samples. Importantly, there was strong concordance between PHB1 concentration curves generated using the QCM-D immunosensor and those from enzyme-linked immunosorbent assay (ELISA), which typically requires 36 h. Our findings illustrate a feasible and reproducible method to detect a bloodborne protein using immunosensor technology that has the potential to improve patient outcomes in sepsis and other serious infectious diseases.

## Graphical abstract

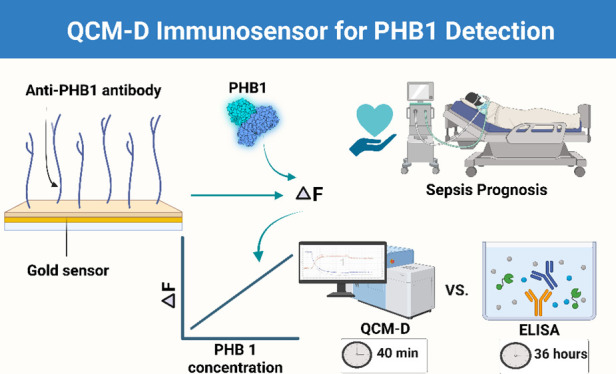

## 1 Introduction

Sepsis is a significant global healthcare challenge owing to its high risk of morbidity and mortality even in countries with advanced medical care.^1–3^ Patient outcomes are further diminished when multiple organ dysfunction syndrome (MODS) occurs. MODS is a serious and often fatal complication of sepsis, characterized by progressive physiological dysfunction in two or more organ systems that is typically reversible.^4^ MODS has also been reported as a leading cause of mortality in other infectious diseases, including severe COVID-19.^5^ While pathologic mechanisms of MODS are complex and multifactorial, it is well-known to involve the release of several immune and vasoactive mediators into the bloodstream.^6–8^ MODS can affect various organ systems, with cardio-pulmonary dysfunction typically preceding renal and hepatic dysfunction.

Consensus findings from critical care specialists indicate that early detection of MODS risk can improve patient outcomes, allowing for prompt initiation of supportive therapy, which will slow deleterious progression.^9–11^ In the case of sepsis-associated MODS, international medical guidelines recommend that fluid resuscitation should commence within the first few hours of diagnosis, concurrent with the administration of intravenous (i.v.) antimicrobials. Severely ill patients will frequently be given iv pressure therapy to optimize cardiac output and mitigate tissue hypoperfusion. Timely intervention is consistently emphasized in the management of sepsis and is a major determinant of outcomes.^12–15^ However, early identification of sepsis patients at high risk for MODS and/or mortality requires addressing two major hurdles: a) validation of reliable and reproducible biomarkers that are associated with increased risk; and b) analytical methods that can rapidly measure these biomarkers, ideally within minutes.

Current bloodborne biomarkers of sepsis severity, such as cyto/chemokines (TNFα, IL-6, IL-8, IL-1RA), endothelial markers (RAGE, MPO), procalcitonin (PCT), and sTREM-1, along with their associated grading systems, often lack the specificity and accuracy required for effective patient management and risk stratification. Our group recently discovered that prohibitin-1 (PHB1) is a plasma protein that sharply increases in concentration within the first 12 h of septicemia.^16^ PHB1 and its isoform PHB2 are relatively small proteins (M.W. 30–34 kDa) that form a ring-like hetero-oligomeric complex within the plasma and mitochondrial inner membranes, where they regulate pleiotropic functions including cellular proliferation, metabolism, and apoptosis. The mechanisms by which PHBs regulate these processes are complex and are known to involve the regulation of growth factor (i.e., EGF, insulin) signaling and mitophagy.^17–19^ In the context of sepsis, a specific role for PHBs has not been fully elucidated but is probably multifactorial as some studies show they help mediate pathogen entry into cells,^20–23^ while others suggest that bloodborne PHBs are associated with mitophagy and inflammatory regulation.^16,24,25^ In any case, preliminary studies from our group using experimental sepsis models and clinical samples show that plasma levels of PHB1 measured ‘early’ in the progression of sepsis (i.e., within the first 12–24 h of symptom onset) are associated with MODS and mortality.

However, until now, plasma PHB1 has only been measured using standard proteomic approaches, including conventional ELISA, which, despite its widespread use in clinical laboratories, typically requires up to 36 h to perform and is costly.^26–28^ The numerous steps and lengthy incubation times required to perform accurate ELISA measurements obviate the utility of this method as a rapid test for MODS risk in critically ill patients.^29–31^ A feasible alternative to ELISA in this context could be the quartz crystal microbalance (QCM), a piezoelectric sensor that measures the resonance frequency variation associated with the mass change on the chip surface. QCM offers several potential advantages compared with ELISA, including versatility in detecting various types of disease biomarkers, relatively short detection times (∼30–60 min), and label-free detection, which reduces the cost and complexity of the assay.^32,33^ QCM with dissipation monitoring (QCM-D) is an extended version that provides additional insights into molecular interactions and layer structures.^34^ This technique has shown great sensitivity in measuring protein adsorption and in thin film characterization.^32,35–37^ Recent studies have demonstrated the clinical potential of QCM-based biosensors for sepsis biomarkers. Pohanka and colleagues developed a QCM-based immunosensor for procalcitonin using nanoparticle-conjugated antibodies and gold electrode surface modification, achieving rapid and sensitive detection in clinically relevant ranges.^38^ These reports suggest that QCM-D technology may offer advantages for the development of an immunosensor that can measure PHB1 as a prognostic biomarker for sepsis and other serious infectious diseases. However, the suitability of the molecular components and their specificity and process parameters, such as sensitivity and rapidness, have to be tested as a first step in this development.

In this study, we (a) developed a QCM-D immunosensor for rapid (i.e., minutes) detection of PHB1 in solution and optimized its basic design parameters; (b) assessed the feasibility and reliability of PHB1 detection and quantification using this immunosensor; and (c) established that the immunosensor could detect PHB1 in clinically relevant plasma concentration ranges.

## 2 Experimental Section

### 2.1 Materials and Reagents

Rabbit recombinant monoclonal prohibitin antibody [EP2803Y] (Abcam, Cambridge, UK) and recombinant human prohibitins-1 and 2 (OriGene Technologies, Rockville, MD, USA) were obtained commercially. Dulbecco’s phosphate-buffered saline (Ca^2+^, Mg^2+^ free) was purchased from Gibco (Thermo Fisher Scientific, Waltham, MA, USA). All other reagents were purchased from Sigma-Aldrich, St. Louis, MO, USA, unless indicated otherwise in the text below.

### 2.2 QCM-D Assembly and Measurement

QCM-D measurements were performed using a Qsense Explorer (Biolin Scientific, Gothenburg, Sweden) and a single flow chamber with a peristaltic pump (Ismatec, Grevenbroich, Germany) at a flow rate of 10, 20, 30, or 150 μL/min. The temperature was kept stable at 20 °C throughout the measurement using the internal control system. Gold-coated quartz crystal sensors (QSX 301, Biolin Scientific) have a fundamental resonant frequency of 5 MHz, as specified by the manufacturer. Hydrophobic polystyrene (PS) QSX 305 sensors were purchased from Biolin Scientific and used without further modification.

### 2.3 Preparation and Cleaning of the QCM-D Sensor

Sensors were carefully cleaned prior to each experiment using a multistep process to ensure optimal performance. Initially, gold sensors were subjected to ultraviolet/ozone (UV/O3) treatment for 10 min followed by a chemical treatment involving a heated 5:1:1 mixture of ultrapure water, ammonia (25%), and hydrogen peroxide (30%) at 75 °C for approximately 5 min. The sensors were then rinsed using ultrapure water and cleaned once more using UV/O3 treatment. After a final wash with ultrapure water, the sensors were dried under filtered air. Hydrophobic PS sensors were immersed in 1% Deconex 11 (Borer Chemie AG, Zuchwil, Switzerland) prepared in deionized water for 30 min at 30 °C. The surfaces were then thoroughly rinsed with deionized water, kept submerged in deionized water for at least 2 h, rinsed with 99% ethanol, and finally dried under filtered air. Each sensor was then mounted in the flow cell, and resonance frequencies were obtained. Following an establishment of a baseline frequency and dissipation under buffer flow, frequency shifts (Δ*F*) and dissipation changes (Δ*D*) were monitored at six overtones, i.e., *i* = 3, 5, 7, 9, 11, and 13. The third overtone, which renders the biggest shifts, was ultimately used as the primary signal for further measurements, while all other overtones yielded qualitatively similar trends.

### 2.4 Antibody Immobilization and Biomarker Binding

The experimental protocol for recombinant PHB1 detection using the QCM-D immunosensor consisted of sequential steps involving protein mixing, exposure to the sensor, and wash procedures, as shown in Figure 1. The isoelectric point of PHB1 lies within the range of 5–6, and all experiments were conducted in PBS buffer at a pH of 7.4. Following the establishment of the baseline frequency in buffer alone, the sensor surface was prepared by introducing the anti-PHB1 antibody (Ab) in solution for adsorption and immobilization, ensuring maximum saturation until frequency and dissipation values reached a steady state. Steady state criteria were met when the values for Δ*F* and Δ*D* did not exhibit drift beyond 1 Hz and 0.2 ppm in the span of 10 min, respectively. The coated sensor was then washed with PBS to remove any loosely bound Ab until a stable plateau was achieved. A solution of PHB1 at varying concentrations was then introduced. All loosely bound molecules were washed at the end with a final rinse in PBS. The total amount of Ab and PHB1 adsorption to the immunosensor was calculated using changes in total Δ*F* after the stable plateau was reached. Specifically, the Δ*F* was determined by averaging the frequency over a 5 min steady-state interval once the signal had stabilized, minus the average of the Δ*F* before introduction of the analyte, as shown in Figure 1. The initial adsorption rate was determined by calculating the slope of the Δ*F* curve during the linear portion of the adsorption process for both the Ab and PHB1 (i.e., prior to plateau or switching to PBS). To ensure the specificity of the immunosensor for PHB1, a control experiment was conducted using recombinant PHB2 under similar buffer conditions.

**Figure 1:**
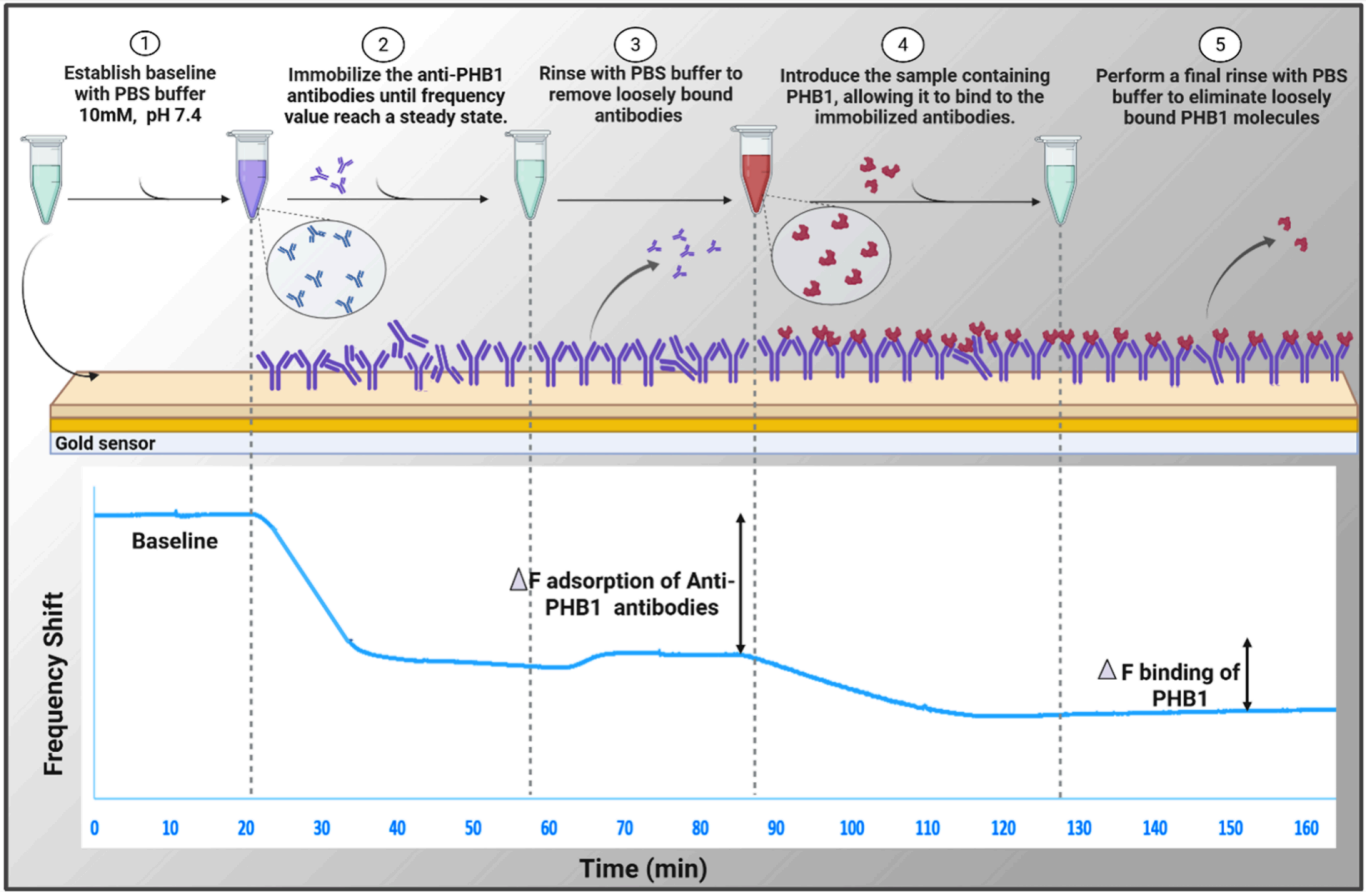
Quartz crystal microbalance with dissipation probe (QCM-D) for real-time monitoring of anti-Prohibitin-1 (anti-PHB1) antibody immobilization and Prohibitin-1 (PHB1) biomarker binding on the gold sensor surface. The lower part of the figure shows real-time shifts in resonance frequency (Δ*F*, Hz) at the flow rate of 20 μL/min. This analysis indicates that the concentration of PHB1 in a sample can be detected in <40 min.

### 2.5 Optimization of Surface Chemistry, Antibody Concentration and Flow Rate for the QCM-D Immunosensor

To optimize the maximum signal within the target conditions of PHB1 detection, that is, the maximum Ab sites that are available for PHB1 binding, systematic adjustment of various parameters was performed. First, treated gold sensors were subjected to a multistep cleaning process recommended by the manufacturer as described above, producing a hydrophilic surface. Polystyrene sensors were used as the hydrophobic surfaces. To corroborate the differing wetting properties of these surfaces, static water contact angle measurements were performed (supplementary Figure S1). Multistep cleaned gold sensors exhibited low contact angles, confirming a hydrophilic surface. In contrast, polystyrene sensors displayed markedly higher contact angles, which verified their hydrophobic nature. Next, Ab concentration effects were studied using two different concentrations (10 and 20 μg/mL) of the Ab in PBS. Lastly, varied flow rates (10, 20, and 30 μL/min) were applied to render different detection times and usage of the solutes. For optimization studies, flow and measurement of the PHB1 samples were continued until a plateau was reached, following which the rinse with PBS was performed.

### 2.6 Establishment of the Standard Curve

Changes in the Δ*F* and Δ*D* induced by PHB1 binding to the QCM-D immunosensor were simultaneously recorded over six overtones. Frequency shifts were calculated as a quantitative measure for different concentrations of PHB1, ranging from 0.25 to 8 μg/mL, which we have empirically determined is the physiological range of the PHB-1 in human and rodent plasma using conventional ELISA methods with the same anti-PHB1antibody. For each concentration, after the immobilization of Ab and rinsing, a flow of the PHB1 solution was maintained for 30 min, following which the rinse with PBS was performed. The measurement for each concentration was repeated two times to build the standard curve of the frequency shift of PHB1 vs known PHB1 concentrations.

### 2.7 Limit of Detection (LOD) and Sensitivity of the Measurements

Sensitivity of the QCM-D immunosensor was evaluated by measuring the frequency shift in response to changes in the PHB1 concentration. The sensitivity was repeatedly tested with different overtone signals in QCM measurements. Concurrently, the LOD was determined through the analysis of the signal-to-noise ratio (SNR), calculated by comparing the amplitude of the desired signal to the amplitude of background noise. Typically, the lowest acceptable SNR for this technology is between 2 and 3. Herein, the LOD was calculated with an SNR of 3, as the pursuit of a reliable detection limit is essential for use of the technology in the clinic.

### 2.8 Enzyme-Linked Immunosorbent Assay (ELISA)

Concentrations of the recombinant PHB1 in PBS were determined by using a quantitative ELISA approach developed by our group. A standard curve for PHB1 was initially established by using purified recombinant PHB1 protein standards and PBS that was serially diluted five times. Samples were added to immunolon-coated 96-well assay plates and incubated overnight at 4 °C. Plates were then washed with PBS+0.05% Tween-20 and blocked for 2 h with 10% fetal bovine serum diluted in PBS. Following the blocking step, the samples were incubated with the anti-PHB1 antibody (1:200 in PBS + 0.05% BSA) for 2 h at 37 °C. After another wash with PBS+0.05% Tween-20, samples were incubated with a secondary antibody (goat antirabbit HRP) for 2 h at room temperature, washed, and then incubated with 10 μM Amplex Red + 100 mU/mL horseradish peroxidase for 20 min at room temperature. Fluorescence values were recorded using a Synergy plate reader (Agilent BioTek, Santa Clara, CA) with a 530ex/590em filter.

## 3 Results and Discussion

In our QCM-D immunosensor, detection is based on antibody–antigen binding rather than enzymatic reactions, making it inherently less sensitive to pH fluctuations compared with enzyme-based or electrochemical sensors.^39^ All measurements were performed under controlled laboratory conditions with the instrument’s built-in temperature control (±0.02 °C), minimizing temperature-induced frequency drift. As the assays were conducted in buffered solutions with a stable pH, no significant performance variation from pH or temperature changes was expected. We first established the feasibility of our immunosensor to bind PHB1. Figure 2A,B presents the QCM-D recordings, which demonstrate Ab adsorption, PHB1 binding, and subsequent washes. Panel A shows the Δ*F* for all overtones. The initial decrease in frequency corresponds to the adsorption of Ab onto the sensor surface. Following a wash step and a modest increase in frequency due to rinsing of the loosely bound molecules, we observed a further Δ*F* decrease upon introduction of PHB1, indicating binding to the immobilized Abs on the sensor. Subsequent wash steps did not significantly alter the frequency, confirming stable PHB1 binding that is expected for a specific Ab-PHB1 binding. Panel B displays the corresponding Δ*D* for all overtones, showing only minimal changes throughout the experiments. This low dissipation variation and minimal spreading of Δ*F* indicated that the adsorbed layers were rigid and thin, allowing the Sauerbrey equation to adequately describe the system. For this reason, and given that Δ*F* provides a direct and reliable measure of mass change without additional assumptions required by viscoelastic models, we chose to report binding responses in terms of Δ*F* rather than converting the data into calculated mass values. It has to be realized that the Δ*F* values are used as a metric of the binding, and any presence of water associated with the layer, as long as it is proportional to the amount of protein, would not change the ratios and correlations. This approach was also consistent with our focus on comparative metrics such as relative percentage error and concordance with ELISA.

**Figure 2:**
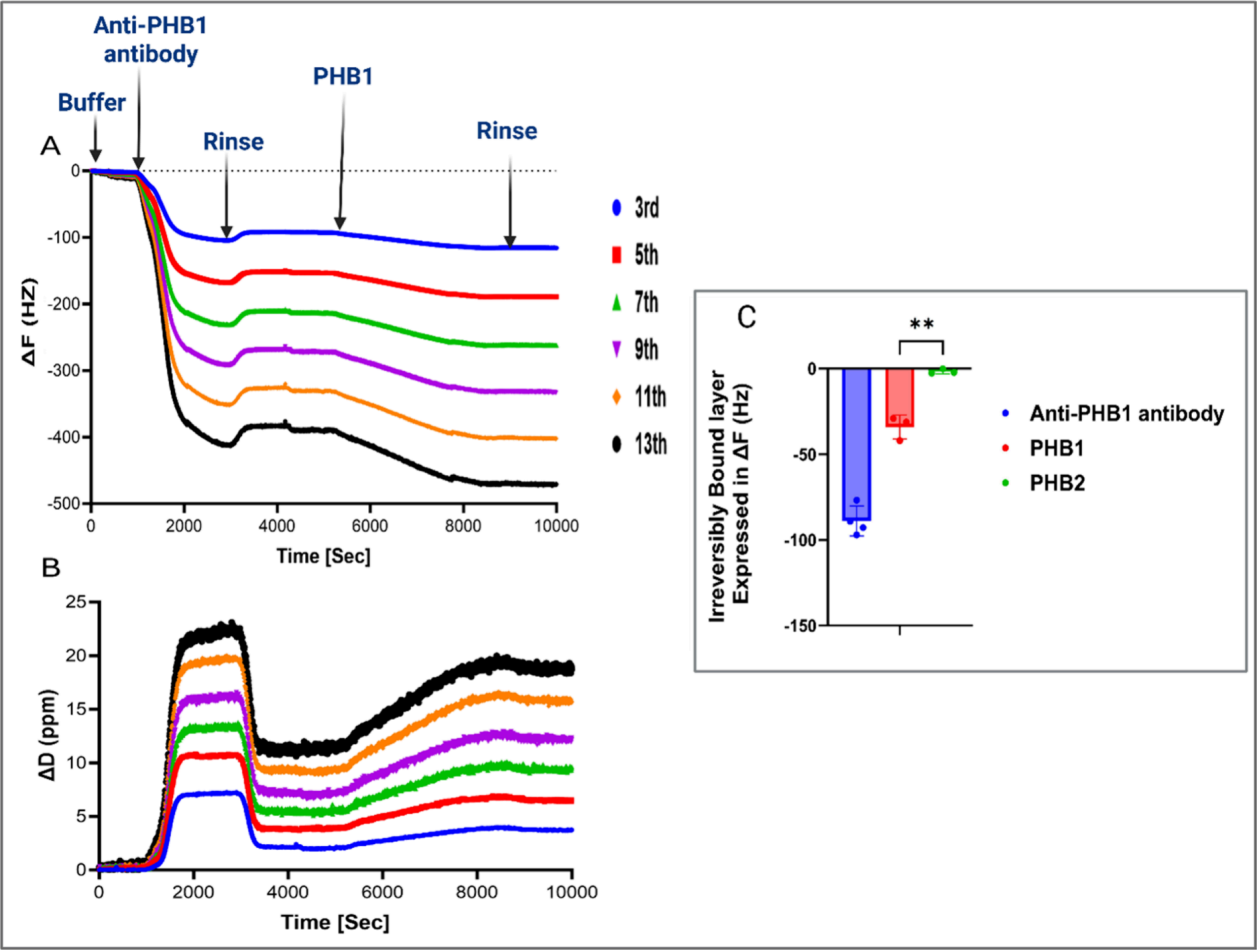
(A,B) Overtone trends depicting Δ*F* and Δ*D* shifts during adsorption of anti-PHB1 antibody (10 μg/mL) and binding of recombinant PHB1 (2 μg/mL), measured at a flow rate of 20 μL/min. (A) QCM-D profile depicting Δ*F* across all overtones tested. (B) QCM-D profile depicting Δ*D* across all overtones tested. (C) The Δ*F*3 shift associated with an irreversibly bound layer of Ab, PHB1 and recombinant PHB2 (2 μg/mL, same flow rate) similar to our biomarker of interest (PHB1) on treated gold sensors. Asterisks (**) indicate statistically significant difference (unpaired two-tailed *t* test, *P* < 0.01) between PHB1 and PHB2.

To test the specificity of our immunosensor for PHB1, we compared the sensor’s response separately to solutions containing only PHB1 or only a closely related, structurally similar isoform (PHB2). Statistical analysis (unpaired two-tailed *t* test) revealed a highly significant difference (*P* < 0.01) in the frequency shifts between response to PHB1 and PHB2, confirming high target selectivity for PHB1, as illustrated in Figure 2C and supplementary Figure S2. The high specificity and affinity of the immunosensor ensure that interactions of interferents in a sample will be minimized. This, in turn, reduces the potential for false-positive results and high noise levels that will adversely impact the sensitivity of measurements.

To enhance the rigor and reproducibility of our immunosensor, we systematically tested its behavior as we varied sensor surfaces, anti-PHB1 antibody concentrations, and flow rates. The Δ*F* associated with the immobilized anti-PHB1 antibody after rinse, and its initial adsorption rate on the treated gold and the polystyrene sensor surfaces, are presented in Figure 3A, with the respective Δ*F* for PHB1 binding on each of the surfaces. The hydrophilic surface of the treated gold sensor exhibited a higher frequency shift and initial adsorption rate during both immobilization of Ab and binding of PHB1, as compared with the polystyrene hydrophobic sensor. Since both frequency shift and initial adsorption rate are related to the total adsorbed mass and rate of adsorption, respectively, an increase in these parameters would mean an increase in the amount of bound antibody and biomarker. The increase in the amount of adsorbed antibody on a hydrophilic surface is likely related to the orientation (i.e., arrangement) of the antibody on the surface, such that more molecules can fit on the surface. These results are in good agreement with previous studies showing that antibody adsorption on hydrophobic surfaces can lead to a less rigid coupling to the surface, affecting antibody orientation.^40^ To further quantify the efficiency of PHB1 biomarker detection relative to antibody surface coverage, we calculated the ratio of frequency shift after PHB1 binding to that after anti-PHB1 antibody immobilization (Δ*F* PHB1/Δ*F* Ab) under each tested condition (Table S1). Notably, this ratio was highest for treated gold sensors (0.38, 0.28) and significantly lower for polystyrene sensors (0.174, 0.193), confirming superior biomarker capture efficiency per immobilized antibody on the gold sensor. Based on these observations, we decided to use the treated sensor because it yields the strongest signal for further experiments.

**Figure 3:**
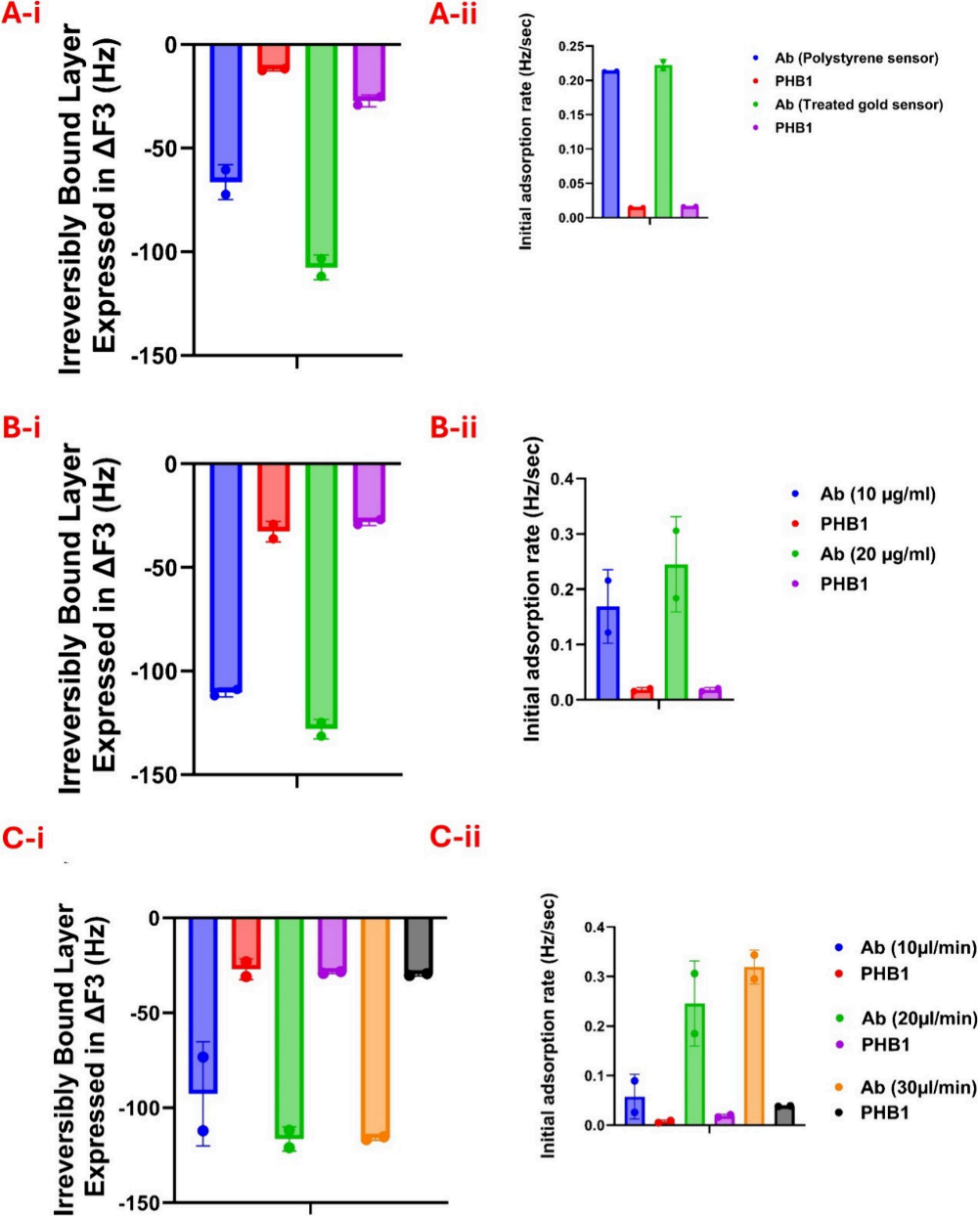
(A-i,ii) Frequency shifts and initial adsorption layer associated with an irreversibly bound layer of anti-PHB1 antibody (Ab) (10 μg/mL) and recombinant PHB1 (2 μg/mL) at a flow rate of 20 μL/min for treated gold sensor and polystyrene sensor; (B-i,ii) Frequency shifts and initial adsorption layer comparing antibody concentrations (10 and 20 μg/mL) for recombinant PHB1 (2 μg/mL) at 20 μL/min; (C-i,ii) Frequency shifts and initial adsorption layer comparing the effect of different flow rates during anti-PHB1 antibody (10 μg/mL) and recombinant PHB1 (2 μg/mL) binding.

When different anti-PHB1 antibody concentrations were compared, a higher frequency shift and initial adsorption rate were observed with the 20 μg/mL concentration, as compared with 10 μg/mL (Figure 3B). These results are consistent with previous reports, which found that at high initial adsorption rates, the surface quickly becomes saturated and the protein diffusion is limited. Consequently, more protein molecules can be accommodated on the same surface area, and the saturation amount increases with increasing concentration of protein in solution.^26,41,42^ However, Δ*F* after PHB1 binding was not changed, suggesting that not all adsorbed anti-PHB1 antibodies were available for PHB1 binding, which may be due to the density and intermolecular interactions and/or orientation of adsorbed antibodies. Moreover, when comparing the ratio (Δ*F* PHB1/ Δ*F* Ab) at different anti-PHB1 antibody concentrations, both 10 μg/mL (0.26, 0.33) and 20 μg/mL (0.215, 0.222) yielded consistent ratios, indicating that higher antibody loading does not linearly translate to increased PHB1 binding likely due to effects such as surface crowding or reduced accessibility (Table S1). Since the signal was not improved with higher antibody concentrations, subsequent studies were performed using a 10 μg/mL concentration.

To determine the impact of flow rate on the immunosensor characteristics, we tested the sensor at three different rates. A flow rate of 10 μL/min enabled detection of PHB1 concentrations within 2 h, while a flow rate of 20 μL/min achieved detection in <40 min, with total consumption of around 800 μL (Figure 1). When the flow rate was increased to 30 μL/min, the sample was flowed until the signal plateaued at around 34 min, resulting in a total consumption of 1020 μL (Figure S3). As shown in Figure 3C-ii, the initial adsorption rate was also assessed. As expected for a mass-transport-influenced system, higher flow rates exhibited higher initial adsorption rates, while lower flow rates resulted in slower initial adsorption. However, higher flow did not alter the final equilibrium binding: the total Δ*F* shift was constant across all conditions (Figure 3C-i), and the Δ*F* PHB1/Δ*F* Ab ratio was similarly consistent (Table S1), confirming that, within the studied range, flow rate influences the adsorption kinetics but not the final extent of binding. Thus, the total Δ*F* shift remains the principal parameter for system evaluation, while kinetic data support our optimization of flow rate and help explain differences in assay time. Taken together, these findings led us to select the flow rate of 20 μL/min as the optimal choice for further development of the immunosensor, as it offers reliable data in under <40 min, which is a reasonable time frame for clinical implementation and at least an order of magnitude faster than ELISA.

To calibrate our immunosensor, Δ*F* responses were recorded for PHB1 concentrations from 0.25 to 8 μg/mL. A strong linear correlation (*R* ^2^ > 0.94) was observed between 0.25 and 4 μg/mL (Figure 4A), whereas extending to 8 μg/mL reduced linearity (*R* ^2^ = 0.92) (supplementary Figure S4). Figure 4B presents representative QCM-D sensogram output curves (frequency shift at the third overtone versus time) for each concentration, further supporting the validity of the calibration and range determination. Accordingly, the analytical linear range was defined as 0.25–4 μg/mL, which our preliminary studies indicate is well within physiological plasma PHB1 levels measured in rodents and humans.

**Figure 4:**
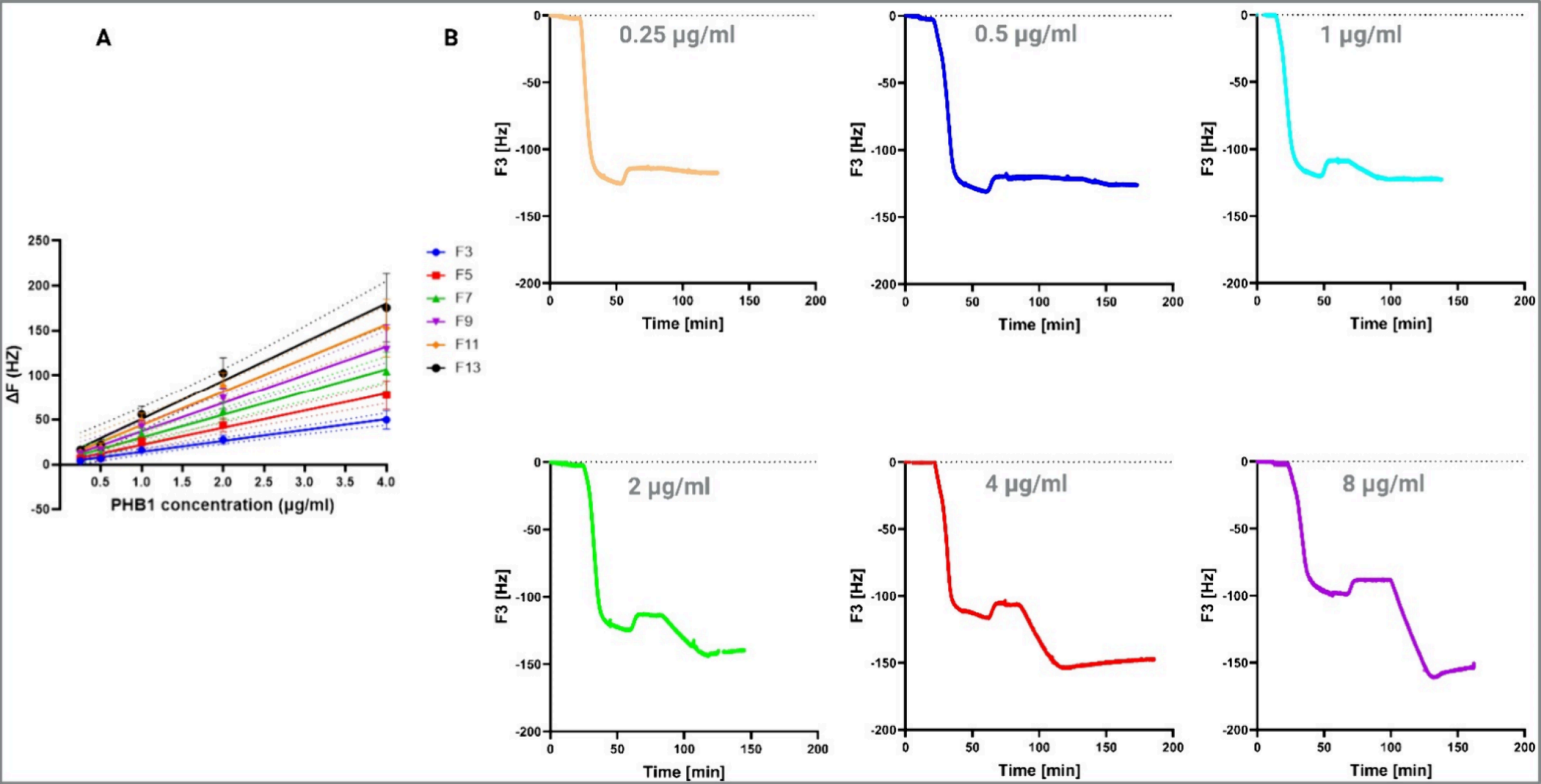
(A) Calibration curve showing the linear relationship between Δ*F* changes resulting from binding of PHB1 concentrations in the 0.25–4 μg/mL range. (B) Representative QCM-D sensograms (time versus frequency shift at the third overtone) for each tested concentration in the 0.25–8 μg/mL range, supporting the analysis of correlation and linearity. For each concentration point, the anti-PHB1 antibody was immobilized and rinsed, and then, a flow of PHB1 solution was maintained for 30 min, followed by PBS rinsing. The resonance frequency shift (Δ*F*) was determined by averaging the signal over a 5 min steady-state interval after the PBS wash, minus the baseline established before PHB1 introduction.

Next, we focused on the sensitivity and LOD of the method within the context of analyzing a clinical sample. LOD was determined by using the linear regression method, considering the signal-to-noise ratio. We plotted the Δ*F* in all overtones against known PHB1 concentrations to obtain a calibration curve (Figure 4A). From this curve, we extracted the slope and the *Y*-intercept. The LOD was then calculated using the following formula: LOD = ((intercept + noise × 3) – intercept)/slope. The lowest detection limit was estimated to be 59 ng/mL for the third overtone. This detection range was well below the physiological plasma levels of PHB1 that our group measured in healthy and diseased patients. As indicated in supplementary Table S2, QCMD offers a relevant detection limit for PHB1 binding across six different overtones. We compared our findings with those of three relevant studies in the field. Zhang et al. reported a low LOD of 2 ng/mL for hepatitis B surface antigen detection using a QCM immunosensor. Their approach involved immobilizing primary anti-Hepatitis B antibodies onto a self-assembled monolayer (SAM) of α-lipoic acid on a QCM gold surface. The key to their enhanced sensitivity was the use of a hyperbranched polymer (HBP) as a bridge to link multiple secondary antibodies, significantly amplifying the detection signal.^43^ In contrast, Minunni et al. developed a QCM aptasensor for HIV-1 Tat protein detection, achieving an LOD of 650 ng/mL, well above the clinically relevant levels of Tat (0.01–10 ng/mL).^44^ In comparison, Pohanka et al. reported a QCM biosensor for procalcitonin with a lower LOD (37.8 ng/L). Their sensor showed enhanced sensitivity attributed to optimization strategies such as nanoparticle signal amplification and gold surface modification.^38^ The LOD of our immunosensor (59 ng/mL) is well within these previous studies using similar technology and, more importantly, is below clinically relevant plasma PHB1 levels. We anticipate that optimization strategies will further improve the sensitivity in future work.

QCM-D sensitivity can be evaluated by measuring the Δ*F* as a function of changes in the PHB1 concentration. The linear relationship between Δ*F* and known concentrations across all overtones (Figure 4A) indicates a robust detection capacity within the range of 0.25 to 4 μg/mL (*R* ^2^ = 0.94). This is consistent with the Sauerbrey equation, which is fundamental to QCM technology and establishes a linear relationship between the resonance frequency of an oscillating quartz crystal and mass changes. It is important to note that for very thin films, variations in the Δ*F* and Δ*D* responses (when divided by the overtone number) are small, indicating that the Sauerbrey equation still gives an adequate description of the system (Figure S5). Our results showed that the sensitivity of the immunosensor at higher overtones is greater than that at lower overtones (supplementary Table S2). These results are in good agreement with previous reports stating that higher overtones (third, fifth, and seventh) in QCM-D measurements showed increased sensitivity due to their shorter penetration depths, allowing for more accurate detection of changes near the sensor surface. In one study, by fitting their data from higher overtones to the Johannsmann model, the authors achieved improved sensitivity for measuring their protein of interest. The study developed a piezoelectric immuno-chip using bacterial cellulose nanocrystals for dengue detection, demonstrating the potential of higher overtone QCM-D for sensitive biomarker analysis.^45–47^

The choice of which overtone to use depends on the specific biomarker characteristics and the clinical context. For biomarkers where the concentration difference between healthy and diseased individuals is small, higher overtones with greater sensitivity may be preferable to detect subtle changes in concentration. For example, our 13th overtone showed the highest sensitivity at 43.38 Hz μg^–1^ ml^–1^. However, for biomarkers present at very low concentrations in the healthy state but greater magnitude difference in the disease state, lower overtones with better limits of detection may be more suitable. In our case, the third overtone had the lowest LOD at 59 ng/mL. This ‘tuning’ flexibility of our QCMD system allows it to be customized for different clinical scenarios, whether the priority is detecting small concentration changes or achieving the lowest possible detection limit for trace biomarkers.

Currently, ELISA is the only validated method for PHB1 quantification in biological samples; therefore, we compared the performance of our QCM-D immunosensor to that of ELISA across a range of PHB1 concentrations. This comparison illustrated that Δ*F* from the QCM-D immunosensor showed strong concordance with those from ELISA across a range of PHB1 concentrations (Figure 5). Pearson correlation analysis demonstrated a very strong, statistically significant linear association between the two methods (*R* ^2^ = 0.9970, *p* < 0.0001), confirming agreement. The average percentage error for both techniques was calculated using the following equation: (standard deviation/average) × 100. Our QCM-D immunosensor demonstrated promising precision with an average percentage error of 12%, compared to ELISA’s 11%. These findings align with previous research on QCM biosensors for sepsis biomarkers. For example, Pohanka et al. (Talanta, 2023) developed a QCM biosensor for procalcitonin detection that employed gold nanoparticle–antibody conjugates and demonstrated full correlation with standard ELISA assays, showing no false positives or negatives across clinically relevant concentrations.^38^ Additionally, these results are consistent with earlier studies using G-QCM (graphene-quartz crystal microbalance) systems, where biomarker measurements from patient sera compared favorably with ELISA.^26^

**Figure 5:**
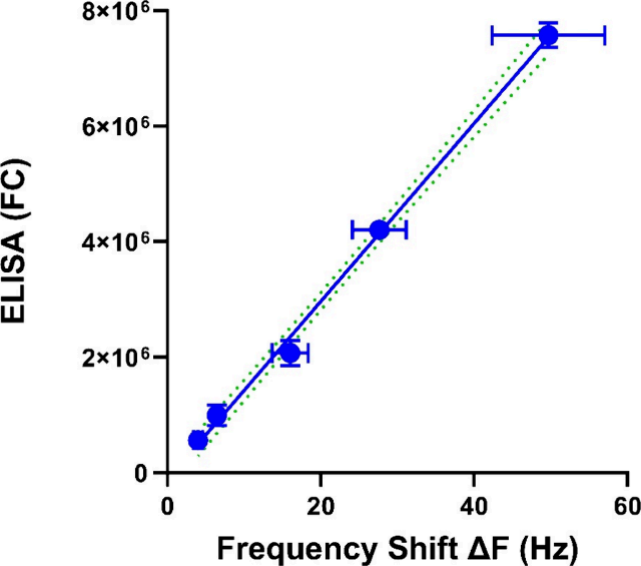
Concordance of our QCM-D immunosensor (Δ*F*) with ELISA fluorescence counts (FC) across the following PHB1 concentrations (250, 500, 1000, 2000, and 4000 ng/mL). Pearson correlation analysis demonstrated a very strong, statistically significant linear association (*R* ^2^ = 0.9970), *p* < 0.0001.

As described above, PHBs are associated with numerous pathophysiological processes involved in the host response to a microbial infection, which is why elevated or dynamic changes in plasma PHB1 levels early in the infection phase may provide a warning of organ injury in critically ill patients.^16,24,25^ The ability of our QCM-D immunosensor to detect PHB1 rapidly (∼40 min) and within the clinically relevant concentration range measured in healthy and diseased patients suggests that it could be integrated into clinical workflows for early risk stratification when patients are admitted to a hospital. In the intensive care or emergency setting, such turnaround times would enable timely clinical decision-making compared to conventional methods (i.e., ELISA), which often require several hours. Thus, the performance demonstrated here not only confirms the analytical feasibility of PHB1 detection using our QCM-D immunosensor but also directly supports its potential role in improving sepsis diagnosis and prognosis. Future work should address nonspecific adsorption and sensor stability with a focus on covalent and highly specific immobilization strategies for antibody attachment to the sensor surface. This will enhance the robustness of the immunosensor when evaluating its performance in experimental models of sepsis using plasma samples. It is also noteworthy that this design prioritizes precise single-analyte quantification of PHB1 and does not support multiplex detection in its current form. Advanced QCM-D approaches that measure multiple molecules in a single assay could be another area of expansion to allow the detection of two or more biomarkers in sepsis patients.^48^

## 4 Conclusions

This study demonstrates the feasibility and potential of QCM-D immunosensor technology for rapid and reliable detection of plasma PHB1, a protein that is strongly associated with infectious diseases and sepsis. Systematic optimization of the gold sensors, anti-PHB1 antibody concentrations, and flow rates in the QCM-D system led to improved performance in sensitivity and LOD. Notably, the application of hydrophilic surface treatments and optimized anti-PHB1 antibody concentrations were efficacious strategies that enhanced PHB1 detection capabilities. The QCM-D immunosensor exhibited analytical sensitivity comparable to that of ELISA, with similar average error rates but dramatically reduced testing time. Linear detection of PHB1 in our QCM-D system ranged from 0.25 to 4 μg/mL, well within the clinically relevant PHB1 plasma concentration ranges. Additionally, the QCM-D system demonstrated high specificity and affinity, highlighting its potential for accurate PHB1 detection. While these initial results are encouraging, it is important to note that this is just the first step in developing a fully functional biosensor, and further development and validation using serum/plasma samples will be necessary. However, the favorable comparison with ELISA, combined with the advantages of lower cost, rapid testing times, and potential for point-of-care use, suggests that our QCM-D immunosensor could be a promising tool for plasma PHB1 detection in the future. Overall, these findings provide the basis for the further development of a robust technology that can identify patients at high risk for MODS early in the progressive course of sepsis (and potentially other severe infectious diseases), allowing them to receive specialized care and close monitoring.

## Supplementary Material



## Notes

Water contact angle comparison of gold sensors; QCM-D sensograms demonstrating specificity, showing distinct resonance frequency shifts (Δ*F*) for PHB1 (2 μg/mL) versus PHB2 (2 μg/mL); optimization sensograms for flow rate; analysis calibration curves; and overtone trends. Table for detection limit, sensitivity data, and table of the calculated ratio (Δ*F* PHB1/Δ*F* Ab) (PDF)

## †

R.N. is a member of the scientific advisory board at Coriolis Pharma, Martinsried, Germany.
