# Supplementary material for: Development of a Piezoelectric Immunosensor to Rapidly Measure Prohibitin‑1 as a Prognostic Biomarker for Sepsis and Other Serious Infectious Diseases
Source: ACS Appl Electron Mater. 2025 Dec 2;7(24):10955–63. doi: 10.1021/acsaelm.5c01783 (PMC12746439; doi:10.1021/acsaelm.5c01783)
Supplement: Supplementary file 1 [file el5c01783_si_001.pdf]

Supporting Information

Development of a piezoelectric immunosensor to rapidly measure prohibitin-1 as a prognostic biomarker for sepsis and other serious infectious diseases

Eman S. Kamel[1], Yusra Rahman[1], Jolonda C. Mahoney[1], Ethan J. Anderson[1,*], Reza Nejadnik[1,*]

[1] Department of Pharmaceutical Sciences and Experimental Therapeutics, College of Pharmacy, University of Iowa, Iowa City, Iowa, 52242, USA.

*** Corresponding authors:**

Reza Nejadnik, email: reza-nejadnik@uiowa.edu;

Ethan J. Anderson, email: ethan-anderson@uiowa.edu

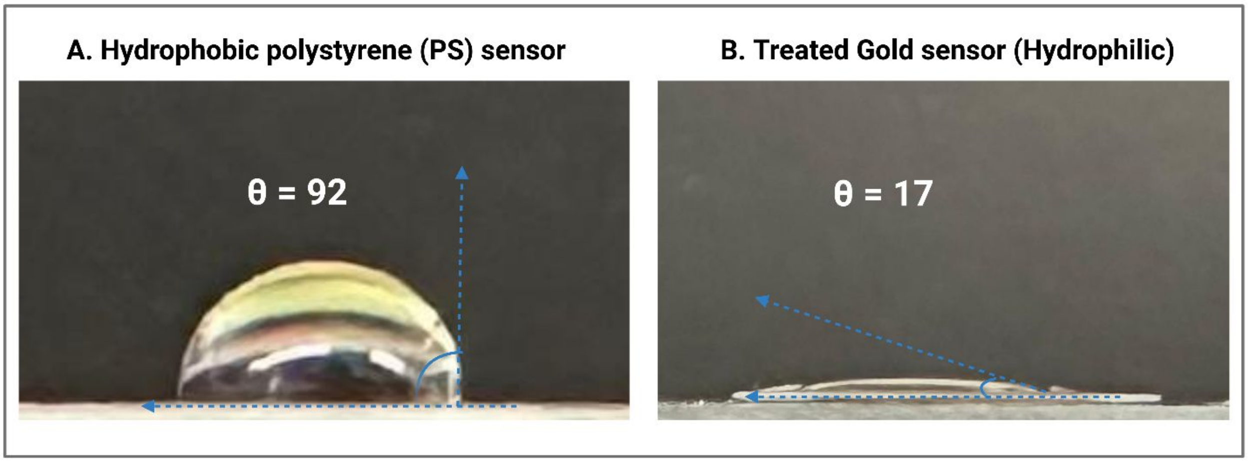

Figure. S1. A drop of water on (A) polystyrene (PS) sensor and (B) treated gold sensor. Water contact angles on the two surfaces are shown.

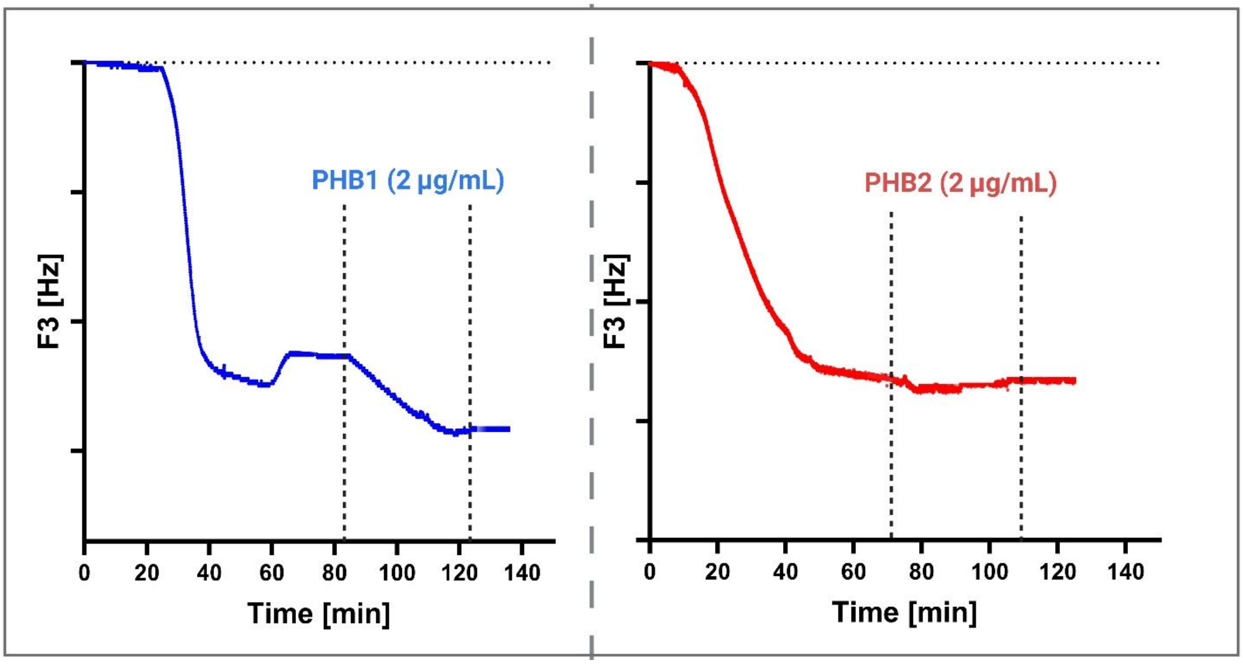

Figure S2. QCM-D sensograms showing resonance frequency shifts (ΔF) for anti-PHB1 antibody (10 µg/mL) functionalized treated gold sensors upon injection of recombinant PHB1 (2 µg/mL, blue trace) and PHB2 (2 µg/mL, red trace) at a flow rate of 20 µL/min.

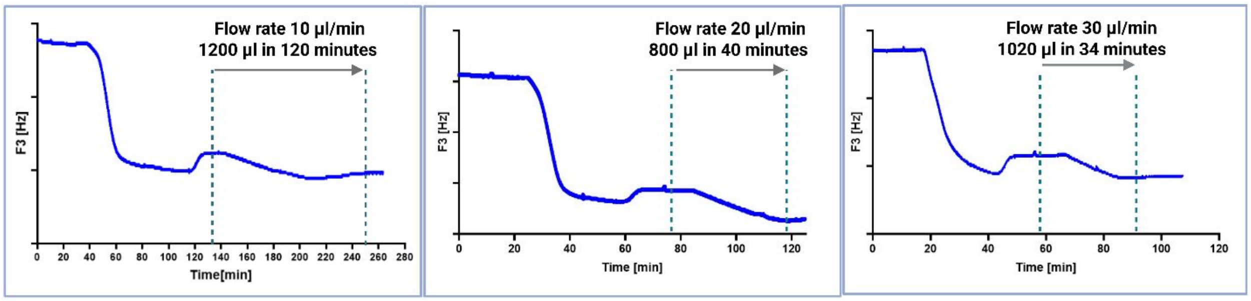

Figure. S3. Sensograms showing frequency shift (ΔF) for QCM-D experiments at three different flow rates (10, 20, and 30 µl/min). Detection time and total sample volume required are indicated for each condition.

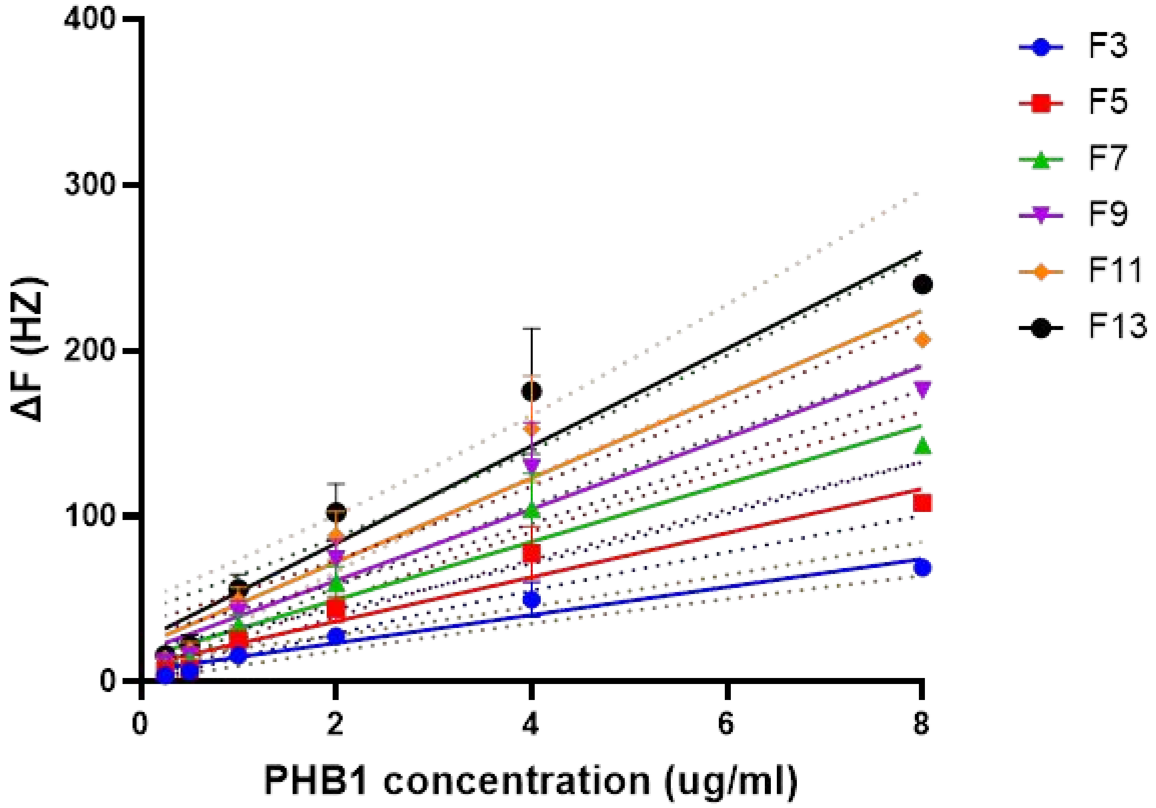

Figure S4. Resonance frequency shifts (ΔF) and PHB1 concentrations in the range of 0.25–8 µg/mL. For each concentration point, anti-PHB1 antibody was immobilized and rinsed, then a flow of PHB1 solution was maintained for 30 minutes, followed by PBS rinsing. The resonance frequency shift (ΔF) was determined by averaging the signal over a 5-minute steady-state interval after the PBS wash, minus the baseline established before PHB1 introduction.

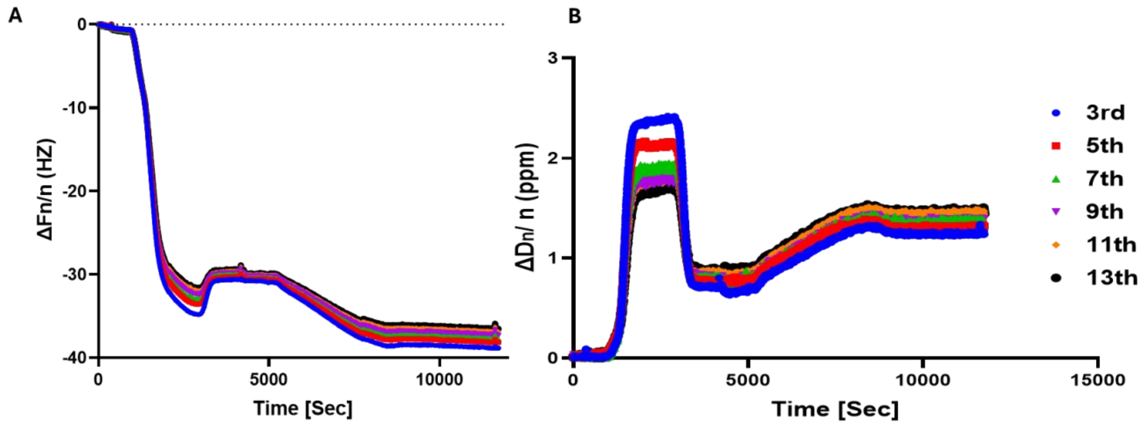

Figure S5. Overtone trends show the ΔF (A) and ΔD (B) during adsorption of anti-PHB1 antibody (10 µg/mL) and binding of recombinant PHB1 (2 µg/mL) at a flow rate of 20 µL/min on a treated gold sensor after dividing by the overtone number.

Table S1. Calculated ratios of initial adsorption rates to frequency shifts (ΔF PHB1 / ΔF Anti-PHB1 antibody) for each experimental condition.

Condition	Ratio (ΔF PHB1 / ΔF Anti-PHB1 antibody)
**Gold sensor (treated) Rep 1**	0.38
**Gold sensor (treated) Rep 2**	0.28
**Polystyrene sensor Rep 1**	0.17
**Polystyrene sensor Rep 2**	0.19
**10 µg/ml Ab (Rep 1, gold)**	0.26
**10 µg/ml Ab (Rep 2, gold)**	0.33
**20 µg/ml Ab (Rep 1, gold)**	0.22
**20 µg/ml Ab (Rep 2, gold)**	0.22
**10 µl/min (Rep 1, gold)**	0.28
**10 µl/min (Rep 2, gold)**	0.32
**20 µl/min (Rep 1, gold)**	0.26
**20 µl/min (Rep 2, gold)**	0.23
**30 µl/min (Rep 1, gold)**	0.25
**30 µl/min (Rep 2, gold)**	0.26

Table S2. Limit of detection and sensitivity values for the different overtones.

Overtone	(Noise*3) (Hz)	LOD (ng/ml)	Sensitivity (Hzμg^{-1}ml)
F3	0.72	59.06	12.19
F5	1.65	86.48	19.08
F7	4.83	188.52	25.62
F9	7.29	229.75	31.73
F11	5.28	140.43	37.60
F13	8.19	189.85	43.14
